# Increased body mass index is a risk factor for acute promyelocytic leukemia

**DOI:** 10.1002/jha2.163

**Published:** 2021-01-06

**Authors:** Sarah M. Kashanian, Andrew Y. Li, Moaath Mustafa Ali, Mark E. Sutherland, Vu H. Duong, Bryan C. Hambley, Kyle Zacholski, Firas El Chaer, Noa G. Holtzman, Mohammad Imran, Ciera L. Patzke, Jonathan Cornu, Alison Duffy, Amy E. Dezern, Ivana Gojo, Kelly J. Norsworthy, Mark J. Levis, B. Douglas Smith, Maria R. Baer, Gabriel Ghiaur, Ashkan Emadi

**Affiliations:** ^1^ Department of Medicine University of Maryland School of Medicine Baltimore Maryland; ^2^ University of Maryland Greenebaum Comprehensive Cancer Center Baltimore Maryland; ^3^ Department of Pulmonary and Critical Care Medicine University of Maryland School of Medicine Baltimore Maryland; ^4^ Department of Emergency Medicine University of Maryland School of Medicine Baltimore Maryland; ^5^ The Sidney Kimmel Comprehensive Cancer Center at Johns Hopkins Baltimore Maryland; ^6^ Department of Pharmacy University of Maryland Medical Center Baltimore Maryland; ^7^ University of Maryland School of Pharmacy Baltimore Maryland; ^8^ Department of Pharmacology University of Maryland School of Medicine Baltimore Maryland

**Keywords:** acute promyelocytic leukemia, APL, BMI, body mass index, obesity

## Abstract

**Introduction:**

Obesity has become increasingly prevalent worldwide and is a risk factor for many malignancies. We studied the correlation between body mass index (BMI) and the incidence of acute promyelocytic leukemia (APL), non‐APL acute myeloid leukemia (AML), acute lymphoblastic leukemia (ALL), and control hospitalized patients without leukemia in the same community.

**Methods:**

Multicenter, retrospective analysis of 71 196 patients: APL (n = 200), AML (n = 437), ALL (n = 103), nonleukemia hospitalized (n = 70 456) admitted to University of Maryland and Johns Hopkins Cancer Centers, and University of Maryland Medical Center.

**Results:**

Patients with APL had a significantly higher unadjusted mean and median BMI (32.5 and 30.3 kg/m^2^) than those with AML (28.3 and 27.1 kg/m^2^), ALL (29.3 and 27.7 kg/m^2^), and others (29.3 and 27.7 kg/m^2^) (*P* < .001). Log‐transformed BMI multivariable models demonstrated that APL patients had a significantly higher adjusted mean BMI by 3.7 kg/m^2^ (*P* < .001) or approximately 10% (*P* < .01) compared to the other groups, when controlled for sex, race, and age.

**Conclusions:**

This study confirms that when controlled for sex, age, and race there is an independent association of higher BMI among patients with APL compared to patients with ALL, AML, and hospitalized individuals without leukemia in the same community.

## 1 INTRODUCTION

The prevalence of overweight and obese individuals in developed western countries has been increasing at an alarming rate throughout the last few decades [1]. In addition to cardiovascular disease, kidney disease, diabetes, and musculoskeletal disorders, the International Agency for Research on Cancer (IARC) has acknowledged 13 cancers associated with overweight and obesity: gastrointestinal malignancies (esophageal adenocarcinoma, and cancers of gallbladder, liver, gastric cardia, pancreas, colon, and rectum), gynecological cancers (ovary, corpus uteri), breast cancer in postmenopausal women, meningioma, multiple myeloma, and thyroid and renal cell cancers [2, 3, 4, 5, 6, 7, 8].

The association between excess body fat and different types of leukemias is not well characterized. In experimental animal models, an inverse association between caloric or dietary restriction and leukemia was observed [8, 9]. A meta‐analysis of 21 prospective cohort studies demonstrated that obesity was associated with increased incidence (relative risk [RR] 1.26, 95% confidence interval [CI] 1.17–1.37, *P* < .001) and mortality (RR 1.29, 95% CI: 1.11–1.49, *P* = .001) of leukemia in adults among all subtypes including acute myeloid leukemia (AML), acute lymphoblastic leukemia (ALL), chronic myeloid leukemia (CML), and chronic lymphocytic leukemia (CLL) [10]. For AML, obesity was associated with an increased incidence, with RR of 1.53 (95% CI: 1.26–1.85, *P* < .001), compared to those with normal weight [10]. Once AML is categorized into acute promyelocytic leukemia (APL) versus non‐APL, patients with APL had a significantly higher body mass index (BMI) than those with non‐APL [7, 11], with the BMI of patients with non‐APL subtypes being similar to that of the general population when adjusted for age, sex, and race [11].

We performed a retrospective analysis of BMIs of patients with newly diagnosed APL, AML (non‐APL subtypes), and ALL at the time of hospital admission, compared with patients without APL, AML, or ALL as a control, admitted to the University of Maryland Greenebaum Comprehensive Cancer Center (UMGCCC), the Johns Hopkins Sidney Kimmel Comprehensive Cancer Center (SKCCC), and the University of Maryland Medical Center (UMMC) between 2002 and 2020.

## 2 METHODS

### 2.1 Study population and design

This two‐center retrospective study was approved by the University of Maryland School of Medicine and the Johns Hopkins School of Medicine Institutional Review Boards. Patients of age 18 years or older with newly diagnosed APL, AML, or ALL based on WHO 2008/2016 criteria [12, 13] initiating chemotherapy at UMGCCC and/or SKCCC between 2002 and 2020 were evaluated. Control patients were of age 18 years or older without a history of APL/AML/ALL admitted (for the first admission) to UMMC from 2015 (when the EPIC electronic medical record was implemented hospital‐wide) to May 2020.

Information on patient demographics including age and height/weight at diagnosis/upon admission, race/ethnicity as reported by patients on admission classified as Caucasian, African American, Hispanic, Asian, Indian, Middle Eastern, or unknown, and sex designated as male or female were collected. BMI was calculated as: weight (kg)/height (m^2^). Patients were classified as underweight (<18.50 kg/m^2^), normal (18.50–24.99 kg/m^2^), overweight (25.00–29.99 kg/m^2^), or obese (≥30.00 kg/m^2^) according to WHO definitions [14].

### 2.2 Statistical analysis

BMI was an independent variable in regression models. Patients with APL were further classified into low, intermediate, or high risk according to the Sanz score [15]. BMI was also compared to Breccia risk score, which predicts overall survival and disease‐free survival, by using the Sanz score as a base, with 0 points for low risk, 1 point for intermediate, and 2 points for high risk; transcript type, 0 for bcr1/2 and 1 for bcr3; *FLT3*‐ITD, 0 for absence and 1 for the presence; morphology, 0 for the classic form and 1 for the variant form; CD34 expression, 0 if absent and 1 if present [16]. Additional baseline characteristics compared with BMI were CD56 and CD15 expression at diagnosis, karyotype t(15;17) alone versus with additional abnormalities, central nervous system (CNS) involvement at diagnosis and relapse.

Descriptive statistics are presented using means with standard deviations and medians, with interquartile ranges for continuous variables and percentages for categorical variables. Continuous variables were compared using *t*‐test or ANOVA. Categorical variables were compared using Pearson chi‐square or Fisher's exact test. Multivariable linear models were used to estimate the adjusted average BMI of the comparison groups. To address potential nonlinearity, we calculated multivariable linear regression models of log‐transformed BMI. Regression model diagnostics were used to check for validity.

To account for missing data, we multiply imputed missing data using the “mi” package [17]. All statistical tests were two‐sided and *P*‐values of less than .05 were considered significant. We used R statistical software (version 4.0.3) for analysis [18].

## 3 RESULTS

### 3.1 Baseline characteristics

The study included 71 196 patients, 200 with APL, 437 with AML, 103 with ALL, and 70 456 others. The median age was 55 years (interquartile range [IQR] 37–68 years), and 35 744 (50.2%) were male. Median BMI was 27.7 kg/m^2^ (range 10.0–145.0 kg/m^2^, IQR 23.8–33.2 kg/m^2^). Racial distribution was 51.3% Caucasian, 41.5% African American, 5.2% unknown, and 1.8% Asian, with other races totaling less than 1% of the study population.

### 3.2 Characteristics of patients subdivided by type

**FIGURE 1:**
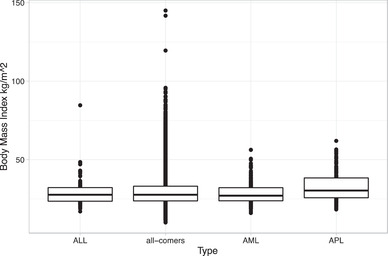
Box plot of unadjusted body mass index against type. Abbreviations: ALL, acute lymphoblastic leukemia; AML, acute myeloid leukemia; APL, acute promyelocytic leukemia

Baseline patient characteristics divided by type are summarized in Table 1. Patients with APL had a significantly higher unadjusted mean (32.5 kg/m^2^, *P* < .001) and median (30.3 kg/m^2^, *P* < .001) BMI than those with AML (28.3 and 27.1 kg/m^2^), ALL (29.3 and 27.7 kg/m^2^), and all‐comers (29.3 and 27.7 kg/m^2^) (Table 1, Figure 1). There was a significant difference in age and sex between the APL, AML, ALL, and other patients as well, with ALL and APL patients younger and APL patients being more frequently female (Table 1).

**TABLE 1:** Baseline characteristics

Baseline characteristics	APL(n = 200)	AML(n = 437)	ALL(n = 103)	All‐comers(n = 70 456)	*P*‐value*
BMI, kg/m^2^, mean (SD)	32.5 (8.8)	28.3 (6.2)	29.3 (8.7)	29.3 (8.1)	<.001
BMI, kg/m^2^, median (range)	30.3 (18.3–62.0)	27.1 (16.0–56.3)	27.7 (17.0–84.7)	27.7 (10.0–145.0)	<.001
Age, years, mean (SD)	49.9 (16.0)	59.3 (15.2)	43.1 (19.0)	53.4 (19.0)	<.001
Sex, male, n (%)	90 (45.0)	244 (55.8)	68 (66.0)	35 342 (50.2)	<.001
Race, n (%)					
Caucasian	107 (53.5)	311 (71.2)	55 (53.4)	36 043 (51.2)	<.001
African American	51 (25.5)	63 (14.4)	25 (24.3)	29 401 (41.7)	
Asian	6 (3.0)	20 (4.6)	5 (4.9)	1264 (1.8)	
Middle Eastern	1 (0.5)	2 (0.5)	1 (1)	3 (0.004)	
Others	16 (8.0)	18 (4.1)	17 (16.5)	62 (0.1)	
Unknown	19 (9.5)	23 (5.3)	0 (0)	3683 (5.2)	

Abbreviations: ALL, acute lymphoblastic leukemia; AML, acute myeloid leukemia; APL, acute promyelocytic leukemia; BMI, body mass index; SD, standard deviation.

* The *P*‐value depicted is for the comparison of all groups together.

### 3.3 Multivariable models

We used multivariable linear regression to estimate the difference in BMI between the subgroups adjusted for gender, age, and race. Patients diagnosed with APL had a 3.0 kg/m^2^ higher adjusted BMI compared to patients diagnosed with ALL (95% CI: 1.08–4.92, *P* = .002) (Table 2). Similarly, patients with APL had a 3.5 kg/m^2^ higher adjusted BMI compared to patients diagnosed with AML (95% CI: 2.16–4.82, *P* < .001), and 3.15 kg/m^2^ higher BMI compared to patients without acute leukemia diagnoses (95% CI: 2.05–4.26, *P* < .001). Compared to all groups, patients with APL had a 3.7 kg/m^2^ higher BMI adjusted for age, gender, and race (95% CI: 2.53–4.88, *P* < .001).

**TABLE 2:** Multivariable analysis of body mass index as dependent variable and sex, age, race, and type as independent variables

	Estimate	Standard error	*T*‐value	*P*‐value
Intercept	32.195	0.892	36.094	<.001
Sex, male	–1.310	0.596	–2.199	.028
Age	–0.043	0.002	–26.189	<.001
Race^a^				
African American	0.697	0.064	10.979	<.001
Asian	–3.457	0.221	–15.633	<.001
Middle Eastern	–2.511	2.937	–0.855	.393
Others	–1.111	0.783	–1.419	.157
Type^b^				
AML	–0.555	0.883	–0.629	.530
APL	3.007	0.980	3.069	.002
All‐comers	–0.883	0.889	–0.993	.321

Abbreviations: AML, acute myeloid leukemia; APL, acute promyelocytic leukemia.

^a^ Caucasian was used as the reference for each race.

^b^ Acute lymphoblastic leukemia was used as the reference for comparison.

On multivariable regression for log‐transformed BMI, patients diagnosed with APL had a 9% higher adjusted BMI compared to patients diagnosed with ALL (95% CI: 3–14%, *P* = .003). Similarly, patients diagnosed with APL had a higher adjusted BMI compared to patients diagnosed with AML (delta: 10%, 95% CI: 6–14%, *P* < .001) and patients without acute leukemia diagnoses (delta: 10%, 95% CI: 7–14%, *P* = < .001) (Table 3).

**TABLE 3:** Multivariable analysis of log‐transformed body mass index as dependent variable and sex, age, race, and type as independent variables

	Estimate	Standard error	*T*‐value	*P*‐value
Intercept	3.454	0.025	136.333	<.001
Sex, male	–0.072	0.002	–36.750	<.001
Age	–0.001	5.28 × 10^−5^	–23.110	<.001
Race^a^				
African American	0.012	0.002	6.346	<.001
Asian	–0.118	0.007	–16.563	<.001
Middle Eastern	–0.071	0.095	–0.745	.456
Others	–0.045	0.024	–1.868	.062
Type^b^				
AML	–0.015	0.028	–0.554	.579
APL	0.090	0.031	2.935	.003
All‐comers	–0.012	0.025	–0.486	.627

Abbreviations: AML, acute myeloid leukemia; APL, acute promyelocytic leukemia.

^a^ Caucasian was used as the reference for each race.

^b^ Acute lymphoblastic leukemia was used as the reference for comparison.

### 3.4 BMI and baseline characteristics among APL patients

In patients diagnosed with APL, we used regression models to check for statistical association between BMI (independent variable) and specific baseline characteristics (dependent variables). None of the univariable models showed statistically significant associations of higher BMI with the examined characteristics including risk status based on Sanz score or Breccia, WBC and platelet counts at diagnosis, *FLT3*‐ITD, CD34/CD56/CD15 at diagnosis, t(15;17) alone versus t(15;17) with additional abnormality, CNS involvement at diagnosis, and relapse status (Table 4).

**TABLE 4:** Univariable analysis of body mass index as independent variable and baseline characteristics as the dependent variables

	Estimate	Standard error	*P*‐value
High risk, based on Sanz^a^	–0.002	0.018	.895
WBC at presentation^b^	–0.335	0.178	.061
Platelets at presentation^b^	–0.110	0.345	.751
FLT3‐ITD mutation^a^	0.010	0.021	.650
CD34 expression^a^	–0.016	0.017	.351
CD56 expression^a^	–0.023	0.026	.380
CD15 expression^a^	–0.034	0.023	.139
Abnormal karyotype^a^	–0.021	0.029	.465
CNS involvement^a^	–0.025	0.065	.701
Relapse^a^	–0.049	0.056	.379
Brecia risk 1|2^c^	–0.646	0.715	.366
Brecia risk 2|3^c^	0.916	0.719	.203

*Note*. Intercepts and standard errors for logistic regression are unexponentiated.

Abbreviations: CNS, central nervous system; WBC, white blood cells.

^a^ Calculated using logistic regression.

^b^ Calculated using linear regression.

^c^ Calculated using ordered logistic regression.

## 4 DISCUSSION

The purpose of this study was to assess the association between elevated BMI and the diagnosis of APL when compared to the non‐APL acute leukemias and nonleukemic population in the same community. BMI of APL patients was significantly higher than those of patients with ALL, AML, and other diagnoses, with 3.2–4.2 and 2.6–3.2 kg/m^2^ higher mean and median values, respectively, in APL patients. Results of this study confirm that when controlled for sex, age, and race, there is an independent association of higher BMI with APL compared with ALL, AML, and other diagnoses.

The data in the current literature on the association of body fat and leukemias are somewhat conflicting, and with this project we tried to clarify this more. Our observation aligns with previous literature describing higher BMI in APL patients compared to AML patients, with BMI of AML patients being similar to that of the general population averages [6, 7, 10, 11]. Of note, other studies have utilized general population averages of BMI as a control; no study has directly used individual data of patients without leukemia admitted to the hospital in the same community as a control. On the other hand, in contrast to prior retrospective studies demonstrating an association between obesity and incidence of all leukemia subtypes (including both chronic and acute leukemias) [10, 19], our study suggests that patients with AML and ALL are overweight but not obese, with BMIs similar to those of the general hospitalized population in the community. Of note, all subtypes of leukemia were included in one group in one of the studies [19], and APL patients were included among AML patients in the other study [10]. Additionally, these studies often included self‐reported height and weight values, which can leave room for error, whereas our study used directly measured values [10, 19].

It has been demonstrated that among the US population, there is a higher proportion of obesity among those 40 years and older compared with those 20–39 years old, with the greatest proportion of obese individuals among non‐Hispanic African Americans [20]. We attempted to mitigate these variations by age and race by correcting BMI for age, race, and sex. Our data suggest that regardless of age/race/sex, APL patients have higher BMI than those with AML and ALL. One limitation of our study is that our data are based solely on hospitalized patients, which may lead to an inherent selection bias; however, based on National Health and Nutrition Examination Survey data, the mean BMI of adults in the United States is 29.1 kg/m^2^ [21], which is similar to the BMI of 29.3 kg/m^2^ found in hospitalized patients in our study.

The reason for the relationship between obesity and APL is unclear. Chronically increased insulin levels have been associated with cancers of breast, colon, pancreas, and endometrium [22, 23, 24, 25, 26]. Obesity can lead to insulin resistance, in turn causing an increase in insulin secretion. Chronic hyperinsulinemia can have tumorigenic effects thought to be due to the direct action of insulin on the insulin receptors in the preneoplastic target cells, or perhaps because hyperinsulinemia causes changes in endogenous hormone metabolism such as the promotion of insulin‐like growth factor‐1 (IGF‐1) [25, 27]. One study in mice demonstrated that increased fat intake leading to weight gain can promote leukemogenesis likely through the IGF‐1 pathway [28]. While some ALL and AML cell lines express insulin and IGF‐1 receptors (IGF‐1R) [29, 30, 31], almost all of APL cell lines such as HL‐60, NB4, and PL‐21 express abundant IGF‐1R protein and proliferate with IGF and IGF analogs stimulation and their growth and basal DNA synthesis decrease with monoclonal antibodies directed against the IGF‐1R and other IGF antagonists [32, 33, 34]. Additionally, the leptin receptor, which is proliferative and bears antiapoptotic effects when activated [35, 36], is selectively upregulated in the APL cells, whereas the normal promyelocytes lack its expression, suggesting a possible link between the leptin‐rich environment in obese individuals and development of APL [35, 36].

In conclusion, we highlight that there is an independent association of higher BMI in patients with APL compared to patients with ALL, AML, and control nonleukemic patients admitted to the hospital in the same community. On the basis of our findings, supported by the prior literature reports, we suggest adding APL to the list of the cancers that are associated with overweight and obesity.

## CONFLICT OF INTEREST

The authors declare that there is no conflict of interest.

## AUTHOR CONTRIBUTIONS

Sarah M. Kashanian contributed to the conception and design of the study, collected and interpreted the data, and wrote the first draft of the typescript. Andrew Y. Li contributed to the conception and design of the study, collected and interpreted the data, and participated in editing and critically reviewing the typescript. Moaath Mustafa Ali contributed in editing and critically reviewing the typescript as well as performing statistical analyses. Gabriel Ghiaur, Vu H. Duong, Maria R. Baer, Noa G. Holtzman, and Firas El Chaer provided critical review and assisted in editing the typescript. Mark E. Sutherland, Ciera L. Patzke, Jonathan Cornu, Alison Duffy, Bryan C. Hambley, Kyle Zacholski, Amy E. Dezern, Ivana Gojo, Kelly J. Norsworthy, Mark J. Levis, B. Douglas Smith, and Mohammad Imran assisted with acquisition of study data. Ashkan Emadi conceived the idea, conceptualized the hypotheses, and designed and supervised all areas of the study. All the authors provided critical feedback, edited, and approved the typescript.
